# The microbial community in decaying fallen logs varies with critical period in an alpine forest

**DOI:** 10.1371/journal.pone.0182576

**Published:** 2017-08-07

**Authors:** Chenhui Chang, Fuzhong Wu, Wanqin Yang, Zhenfeng Xu, Rui Cao, Wei He, Bo Tan, Meta Francis Justine

**Affiliations:** 1 Long-term Research Station of Alpine Forest Ecosystems, Institute of Ecology & Forestry, Sichuan Agricultural University, Chengdu, PR China; 2 Collaborative Innovation Center of Ecological Security in the Upper Reaches of Yangtze River, 8 Chengdu, China; USDA Forest Service, UNITED STATES

## Abstract

Little information has been available on the shifts in the microbial community in decaying fallen logs during critical periods in cold forests. Minjiang fir (*Abies faxoniana*) fallen logs in decay classes I-V were in situ incubated on the forest floor of an alpine forest in the eastern Tibet Plateau. The microbial community was investigated during the seasonal snow cover period (SP), snow thawing period (TP), early growing season (EG) and late growing season (LG) using Phosphorous Lipid Fatty Acid (PLFA) analysis. Total microbial biomass and microbial diversity in fallen logs were much more affected by critical period than decay class, whereas decay class had a stronger effect on microbial diversity than on microbial biomass. Abundant microbial biomass and microbial diversity in logs even without the cover of snow were observed in winter, which could not be linked to thermal insulation by snow cover. The freshly decayed logs functioned as an excellent buffer of environmental variation for microbial organisms during the sharp fluctuations in temperature in winter. We also found distinct decay patterns along with seasonality for heartwood, sapwood and bark, which requires further detailed research. Gram^-^ bacteria mainly dominated the shifts in microbial community composition from SP to EG, while fungi and Gram^+^ bacteria mainly dominated it from SP to TP. Based on previous work and the present study, we conclude that fallen logs on the forest floor alter ecological processes by influencing microbial communities on woody debris and beneath the soil and litter. Our study also emphasizes the need to maintain a number of fallen logs, especially fresh ones, on the forest floor.

## Introduction

Fallen logs are major structural features in virgin forests. Fallen logs in woodlands play vital roles with many ecological functions, such as sequestering carbon, providing a habitat for organisms, nursing tree seedlings and influencing soil and sediment transport and storage [1,2]. Decaying fallen logs can also be a major long-term source of energy and nutrients in the forest system. The decomposition of fallen logs as a mechanism of carbon and nutrient release and energy flow is a crucial process that connects forests with the soil and atmosphere. It is well known that the microbial community participates substantially in the continuous process of the coarse woody debris (CWD) decomposition process [3–5], and the microbial community is controlled by substrate quality changes, environmental fluctuations and their interactions. Shifts in the microbial community are also an external indication of decomposition conditions. Consequently, knowledge about microbial community shifts in decaying fallen logs will provide essential evidence for a detailed understanding of the process of CWD decomposition.

Theoretically, the microbial community in decaying fallen logs consists of ubiquitous bacteria, micro-eukaryotes, non-decay fungi and wood-decay fungi [4]. The dominant microbial groups participating in wood decay may varied with critical periods and decay stage, because of the distinct morphologies, growth strategies, and ecological niches of different microbial groups [6,7]. Fungi are considered as the most important participant in degrading recalcitrant ingredients. Fukasawa et al. [8] indicated that the decay process of CWD can be divided into two phases characterized by different dominant organic chemical constituents, and the dominant fungal taxon in each phase is also diverse. Compared to fungi and Gram^-^ bacteria, Gram^+^ bacteria are more efficient in degrading organic carbon, whereas the former two depend strongly on fresh input of organic material [9]. Therefore, an investigation of the changes in microbial community composition in decaying fallen logs can provide key clues for understanding the mechanisms of log decomposition. To date, little information is available on the changes in microbial community structure in decaying fallen logs at different critical periods.

CWD decay is a long-term process in timescales, which lasts for several decades, even up to centuries for complete wood decay in nature. As a result, investigating the temporal dynamics of the microbial community in decaying fallen logs looks unachievable. Fortunately, CWD in various decay stages are left on the forest floor, which provides important materials for revealing the decay process of CWD [8,10,11]. Significant differences in physical structure and chemical composition have been documented between fresh and well-decayed wood [12,13], resulting in different microbial diversity and functional diversity in decaying wood and other plant debris, and soil beneath the wood [14–16]. Thus, a method of direct observation in combination with chronosequence approach might be used to measure microbial community succession in decaying fallen logs.

Temperature and moisture are closely related to the respiration rate and microbial activity in CWD, and the interactive effect varies with seasonal fluctuations [17,18]. Annually, a cold biome experiences an extremely cold winter and a temperate growing season, resulting in sharp fluctuations in environmental temperature and moisture and a related microbial community shift. In the high-frigid forest ecosystem, fallen logs with different decay classes on the forest floor are directly exposed to an air environment, which accumulates less snow and experiences more frequent fluctuations in temperature and moisture during cold winters. Accordingly, the biomass, dominant microbial groups and the diversity of the microbial community in decaying fallen logs might vary greatly with critical periods. In contrast, well-decayed fallen logs might be more sensitive to seasonal fluctuations resulting from a more porous physical structure and high water capacity. Consequently, measurements of the structure of the microbial community in different decay classes of fallen logs at different critical periods will enhance understanding of the role that the microbial community played in decaying fallen logs.

The alpine forest located in the upper reaches of the Yangtze River and the eastern Qinghai-Tibet Plateau plays important roles in holding fresh water, sequestering carbon and indicating climate change [19,20]. Previous studies have indicated that fallen logs with different decay classes account for 53.00 t·ha^-1^ in the primary Minjiang fir (*Abies faxoniana*) forest ecosystem [21] and play crucial roles in nurturing biodiversity, maintaining site productivity and holding water [22]. However, the changes that occur in the microbial community in different decay classes of fallen logs with critical periods remain unknown. Therefore, Minjiang fir fallen logs of different decay classes were in situ incubated over one year, and a series of “microbial community snapshots” were obtained at four critical periods. Our main objectives were to assess (1) the seasonal dynamics of microbial community structure in fallen logs of a range of decay classes and the response of dominant microbial groups to seasonal fluctuations; and (2) the contribution of each factor (critical period and substrate quality) affecting the microbial community across seasons.

## Materials and methods

### Ethics statement

Western Sichuan Forestry Bureau to conduct scientific experiments in the Bipenggou Nature Reserve since March 2006. And the person in charge is Weiquan Wen. The fallen logs collected for this study were only sampled at a very limited scale, and thus had negligible effects on broader ecosystem functioning. Moreover, this research was carried out in compliance with the laws of the People’s Republic of China. The research did not involve measurements of humans or animals and no endangered or protected plant species were involved.

### Study sites

This study was conducted at the Long-term Research Station of Alpine Forest Ecosystem (102°53′-102°57′ E, 31°14′-31°19′ N, altitude 2458–4619 m), which is located at the transition area from the Tibet Plateau to the Sichuan Basin in Southwest China. The mean annual precipitation is approximately 850 mm, with the peak occurring in the growing season. The mean annual temperature ranges from 2 to 4°C, with minimum and maximum temperatures of -18 and 23°C, respectively. The seasonal freeze-thaw cycle begins in early November after the first snowfall, and the soil remains frozen until April of the next year [23]. The snow data have been published by He et al. [24]. The Minjiang fir (*Abies faxoniana*) forest is the dominant forest type in the alpine zone, and the canopy layer is mainly composed of Minjiang fir and *Sabina saltuaria*, *Salix paraplesia*, and *Fargesia nitida*; the shrub layer is mainly dominated by *Rhododendron lapponicum*, *Berberis sargentiana*, and *Sorbus rufopilosa*; and the herb layer is mainly composed of *Rosa sweginzowii*, *Cacalia* spp., *Cystopteris montana*, *Carex* spp., and *Cyperus* spp.

### Design of the decomposition study

Our new method of estimating long-term wood decay requires the collection of fallen logs at various stages of decay. The decay stage was classified on the following scale of I-V according to the modified criteria of Rouvinen [25,26].

- DC1: Died less than one year before sampling, cambium still fresh, no moss or other vegetation present.
- DC2: Bark mostly intact, cambium decayed, minimal moss/vegetation.
- DC3: Bark sloughing or absent, heartwood mostly sound, minimal moss/vegetation.
- DC4: Bark almost absent, heartwood rotten and does not support its own weight, covered with moss/vegetation.
- DC5: Bark absent, covered in thick moss vegetation, partially collapsed.

Freshly cut trees were used so that all added wood was of a uniform decay class (DC1). For logs in DC1-3, the species is visually recognizable, while it is not in DC4-5. Therefore, help from experienced forest workers is of particular importance. In August 2013, 45 logs of *Abies faxoniana* that were 120–150 cm in length and 35±5 cm in diameter were collected from the primary Minjiang fir forest for the experiment.

Based on our previous field investigation, logs in decay class I-V were in situ incubated in three permanent plots 100 m × 100 m in size and at least 500 m apart. These three permanent plots were established in three representative Minjiang fir forests (31.23° N, 102.88° E, 3582 m a.s.l.). In each sampling plot, one forest canopy measuring 25 m × 25 m was positioned with similar canopy density, homogeneous aspects and slopes to avoid heterogeneity in topography and climate while maintaining natural experimental conditions.

We transported the selected logs to the incubation plot separately, minimizing any damage to the bark and epiphytes existing on the logs, which we considered a key component for subsequent experiments. In particular, logs in advanced decay classes (DC4-5) are easily destroyed during transportation due to their soft and decaying nature, so we placed them in the nearest plots that met the plot setting requirements. Within each subplot, the logs should be in the same compass orientation. Thus, between August 10 and 15, 2013, all the logs were placed in their final position in their respective incubation sites. Within each subplot, the logs were positioned on the soil surface 30 cm apart, making good contact with the soil.

We are continuing to conduct a long-term in situ fallen log decomposition experiment, but here we focused on the dynamics of the microbial community in fallen logs in decay class I-V at different critical periods.

### Sampling

According to our previous research and long-term sequential temperature observations, we divided the entire year into the seasonal snow cover period (SP), snow thawing period (TP), early growing season (EG) and late growing season (LG). We began the sampling on February 26, 2014 (SP) after 195 days restoration and on April 23, 2014 (TP), August 20, 2014 (EG) and October 22, 2014 (LG). Adjacent to each end of a stem section (log), a 2-cm-thick disk was sawn out for analyses of wood samples at DC1-3. For advanced decayed deadwood, adequate fragments were collected. Each anatomical structure (heartwood, sapwood and bark) was sorted separately for lab analysis.

**Fig 1:**
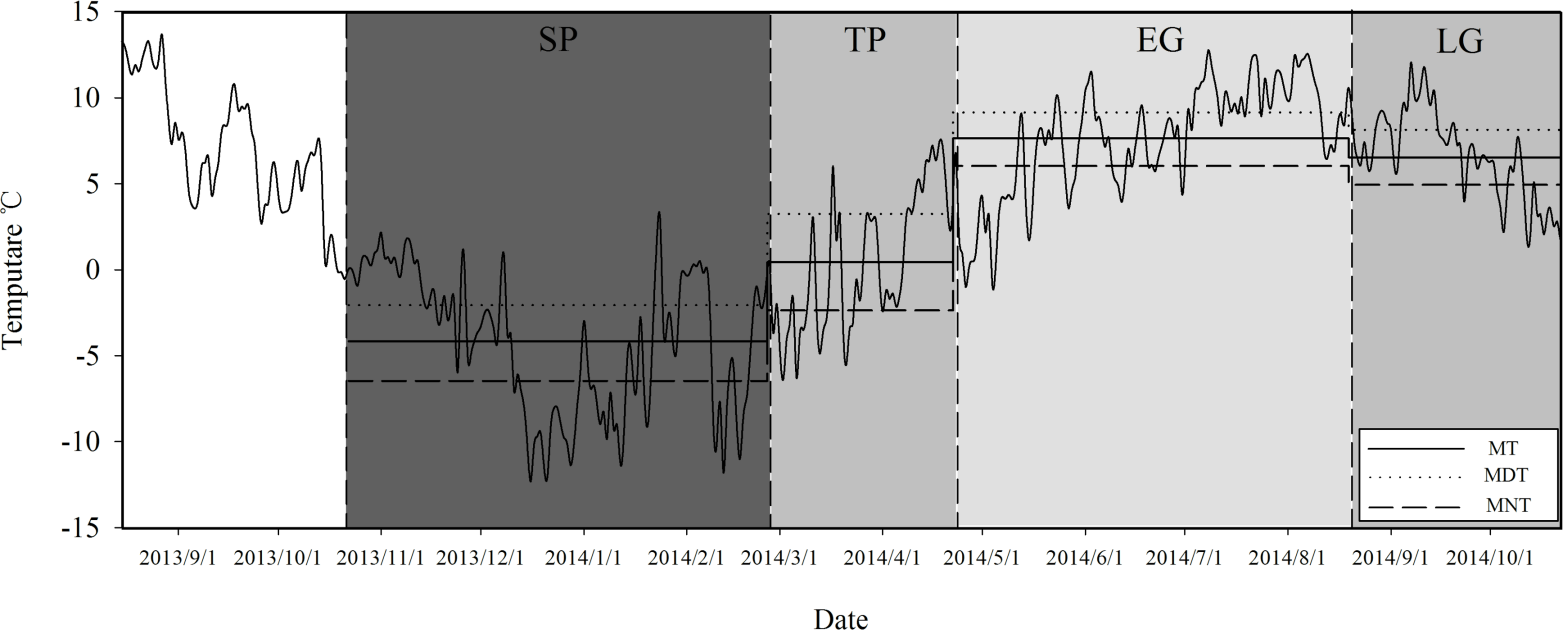
Dynamics of daily mean temperature (°C), mean temperature (MT, °C), mean daytime temperature (MDT, °C), and mean nighttime temperature (MNT, °C) in the atmosphere in an alpine fir forest in the eastern Qinghai-Tibet Plateau from August 15, 2013 to October 22, 2014. SP seasonal snow cover period, TP snow thawing period, EG early growing season, LG late growing season.

The air temperature (Fig 1) was measured at 2-h intervals over the entire study period (August 15, 2013—October 22, 2014) using iButton DS1923-F5 Recorders (iButton DS1923-F5, Maxim/Dallas Semiconductor, Sunnyvale, USA), which was placed in one litterbag (nylon, 20×20 cm, mesh size 0.5 mm) hung on a tree in each subplot to protect it from direct sunshine and precipitation. To characterize the temperature dynamics of each critical period, we also calculated the mean temperature (MT), mean daytime temperature (MDT), and mean nighttime temperature (MNT), respectively (Fig 1).

### Data collection

#### Moisture content

The moisture content of each sample was measured (Table 1). For this analysis, fresh subsamples were weighed (M_1_) and subsequently oven-dried for 24 h at 60°C (M_2_). The moisture content (MC, %) was calculated as MC = (M_1_-M_2_)/M_1_.

**Table 1:** Seasonal changes in the moisture content of three fallen log components (heartwood, sapwood, and bark) in different decay classes (decay classes 1–5 for heartwood and sapwood, decay classes 1–3 for bark) at each critical period in the alpine fir forest of the eastern Qinghai-Tibet Plateau. The data are presented as the means (SE, n = 3). SP: seasonal snow cover period, TP: snow thawing period, EG: early growing season, LG: late growing season.

Components	Decay Classes	SP	TP	EG	LG
Heartwood	Ⅰ	20.7(2.3)	43.1(9.2)	67.5(1.5)	34.1(0.4)
	Ⅱ	48.7(3.6)	36.9(2.4)	58.6(3.0)	50.1(3.3)
	Ⅲ	35.3(13.8)	63.9(0.0)	59.4(7.2)	54.0(13.3)
	Ⅳ	74.0(3.6)	78.4(0.3)	78.2(3.6)	17.7(4.2)
	Ⅴ	77.4(1.8)	47.8(5.8)	70.2(0.6)	28.7(0.0)
Sapwood	Ⅰ	38.6(3.7)	38.8(0.3)	68.7(1.3)	31.2(0.5)
	Ⅱ	22.8(0.8)	39.7(8.5)	63.3(1.6)	32.6(0.3)
	Ⅲ	17.4(1.1)	51.0(2.4)	54.7(2.0)	60.1(7.8)
	Ⅳ	49.6(2.5)	69.3(2.9)	80.3(0.2)	21.3(0.2)
	Ⅴ	52.9(0.8)	52.2(0.0)	64.2(0.3)	27.8(0.0)
Bark	Ⅰ	27.4(0.5)	45.4(3.4)	61.2(2.2)	41.4(1.6)
	Ⅱ	11.4(0.5)	29.6(4.1)	53.6(5.1)	59.5(1.8)
	Ⅲ	23.0(1.1)	28.1(5.3)	46.2(4.6)	54.7(0.0)

#### PLFA extraction

Samples for microbial PLFA extraction were immediately freeze-dried and stored at -70°C. Lipid extraction was performed according to previous protocols [27]. Briefly, the phospholipid fraction was extracted and converted to fatty acid methyl esters (FAMES) [28]. The FAMES were separated, quantified, and detected with a SHIMADZU gas chromatograph (GC) equipped with a mass spectrometer (QP2010-Ultra) and a GC column (Cat NO. 13623) and controlled by an Operation System with reference to standards. FAME standards were obtained from Supelco (Bacterial Acid Methyl Ester Mix, 47080-U; 37 Component Fatty Acid Methyl Ester Mix, CRM47885). For an internal standard, methyl nonadecanoate (C19:0) was obtained from Supelco (74208-1G). More details please see http://dx.doi.org/10.17504/protocols.io.ixmcfk6.

The PLFA biomarkers were used to determine the fungal population (18:2ω6c), the bacterial population (sum of 15:0, i15:0, a15:0, 16:0, 16:1ω5t, 16:1ω7c, 16:1ω9c, 17:0, a17:0, i17:0, cy17:0, 18:1ω7c, and cy19:0), and the microeukaryotic (Micro) population (sum of 18:3 and 20:4). The Gram^+^ population was the sum of i15:0, a15:0, i17:0, and a17:0. The Gram^-^ population was the sum of 16:1ω7c, 16:1ω9c, cy17:0, 18:1ω7c, and cy19:0 [9,29].

### Data analysis

**Fig 2:**
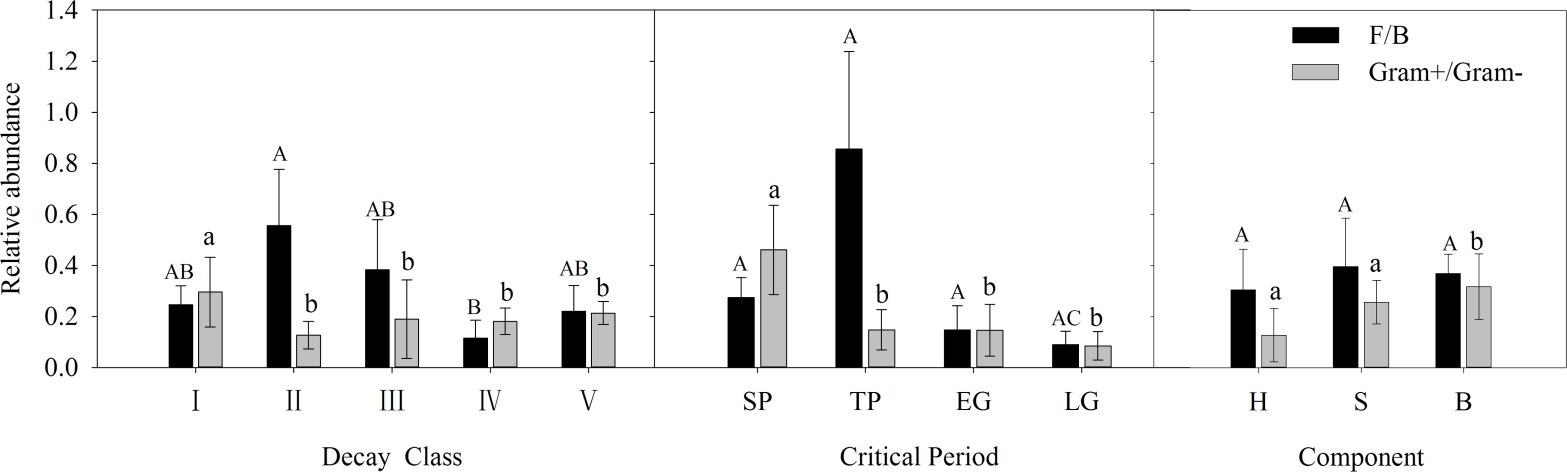
Averaged relative abundance of fungi to bacteria (F/B) and Gram^+^ bacteria to Gram^-^ bacteria (Gram^+^/Gram^-^) of the five decay classes (I-V), four critical periods (SP, TP, EG, and LG), and three components (heartwood, sapwood, and bark) in the alpine fir forest of the eastern Qinghai-Tibet Plateau. The data are presented as the means (SE, n = 3). Values are square root transformed. Different capital letters indicate significant differences within treatments at the 0.05 level (F/B). Different lowercase letters indicate significant differences within treatments at the 0.05 level (Gram^+^/Gram^-^) SP: seasonal snow cover period, TP: snow thawing period, EG: early growing season, LG: late growing season, H: Heartwood, S: Sapwood, B: Bark.

The ratios of F/B and Gram^+^/Gram^-^ were calculated by PLFA biomass to determine differences in microbial community composition between components, decay classes and critical periods (Fig 2).

The α- and β-diversity of the microbial community were calculated based on the number of occurrences of individual PLFA as follows. α-diversity, the PLFA signature richness in each decay class, was expressed as the average occurrence number of individual PLFA signatures per log in each decay class. β-diversity is an approach [30] to the dissimilarity of PLFA signature compositions between logs. The computer program EsimateS 9 [31] was used to calculate the Sørensen similarity index (C_*s*_) as described by Fukasawa [14]:

$$C_{\text{s}}=2j/(a+b)$$ (1)

where *j* is the number of individuals occurring in both logs, a is the number of individuals in log A, and b is the number of individuals in log B. C_*s*_ is set to equal 1 in cases of complete similarity and 0 in cases of complete dissimilarity.

**Fig 3:**
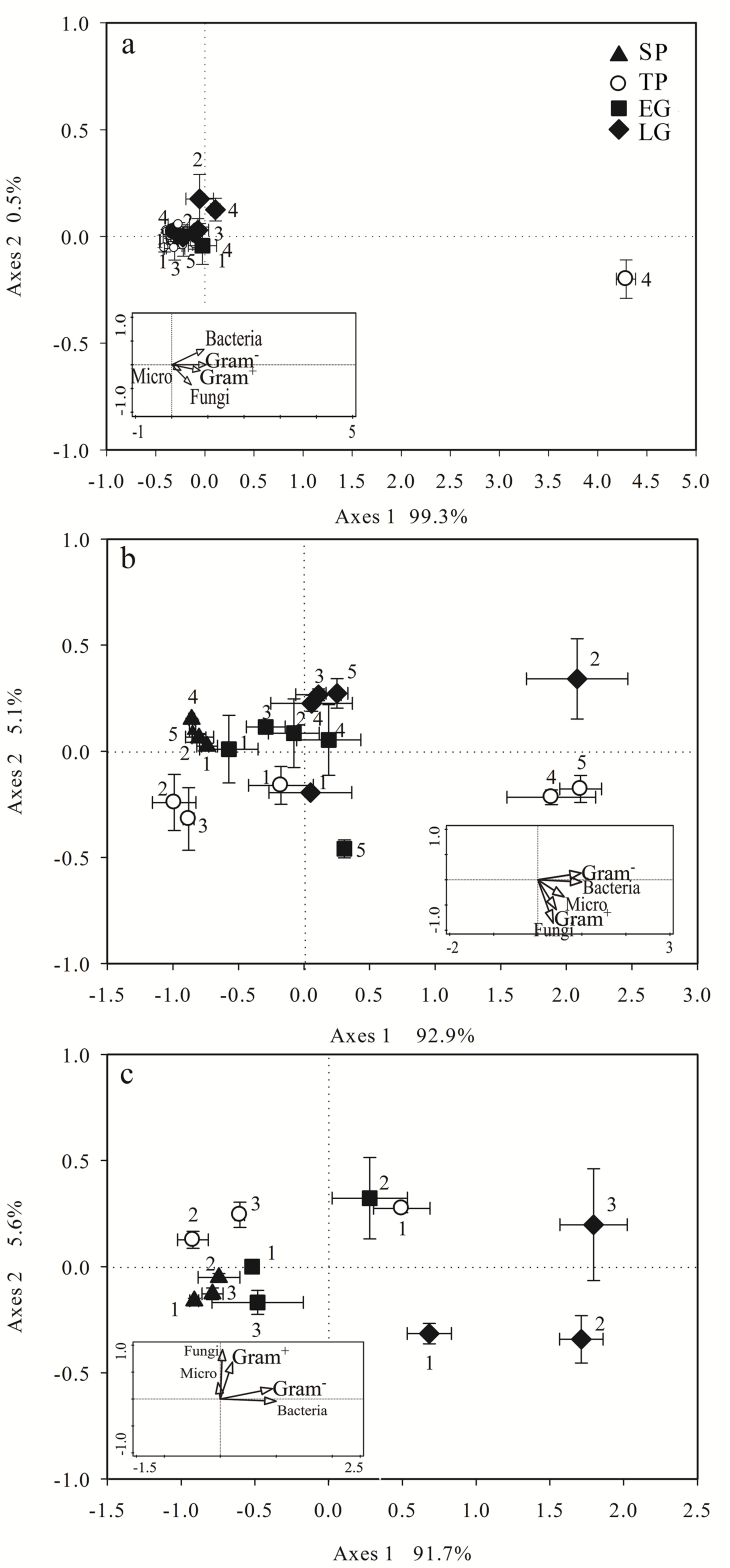
**NMDS plot of PLFA profiles for heartwood (a), sapwood (b) and bark (c) collected at four critical periods in the alpine fir forest of the eastern Qinghai-Tibet Plateau.** Error bars represent standard error (n = 3). Inserted plot shows the relationship between a set of variables (in this case, critical period and decay class) and taxonomic groups. The angle and length of the vector indicate the direction and strength of the variable and the NMDS axis.

Shifts in the taxonomic microbial community structure of logs were observed as indicated by two-dimensional ordination plots using nonmetric multidimensional scaling (NMDS) of PLFA groups (Fig 3). Summarized pattern was used as the sum of square root-transformed PLFA monomers. Within the plots, the closer the points, the more similar they were in microbial community structure. The length of the projection distance from points to vector indicates the abundance of variables (the inserted plot in Fig 3). The NMDS analyses were conducted using Canoco for windows (version 5.0) and Sigmaplot (version 12.5) for post-production.

One-way ANOVA was used to test for significant differences (*P*<0.05) in F/B, Gram^+^/Gram^-^ values between critical periods, decay classes, and components. A repeated-measures analysis of variance (ANOVA) was completed to test the effects of period, decay class, and their interactions on total PLFA (PLFA_tot_) biomass and bacterial, fungal, Gram^+^, Gram^-^, Micro PLFA biomass, α- and β-diversity over time. All the ANOVA analyses were conducted using SPSS (version 20.0).

## Results

### Microclimate

Both MDT and MNT were subzero in SP and above zero in EG and LG during the investigation (Fig 1), while the critical period of TP experienced the largest temperature fluctuation between day and night. Consistent with the precipitation in this region, all the log components also experienced the highest moisture content in EG (Table 1). The lowest moisture content was most appeared in SP, few was appeared in LG. Pronounced higher moisture contents in well-decayed (DC4-5) heartwood and sapwood than in less decayed ones were observed in both SP and EG. Conversely, bark samples of DC1 always held a higher moisture content than samples of DC2-3 throughout the whole-year incubation except in LG.

### Microbial biomass

**Fig 4:**
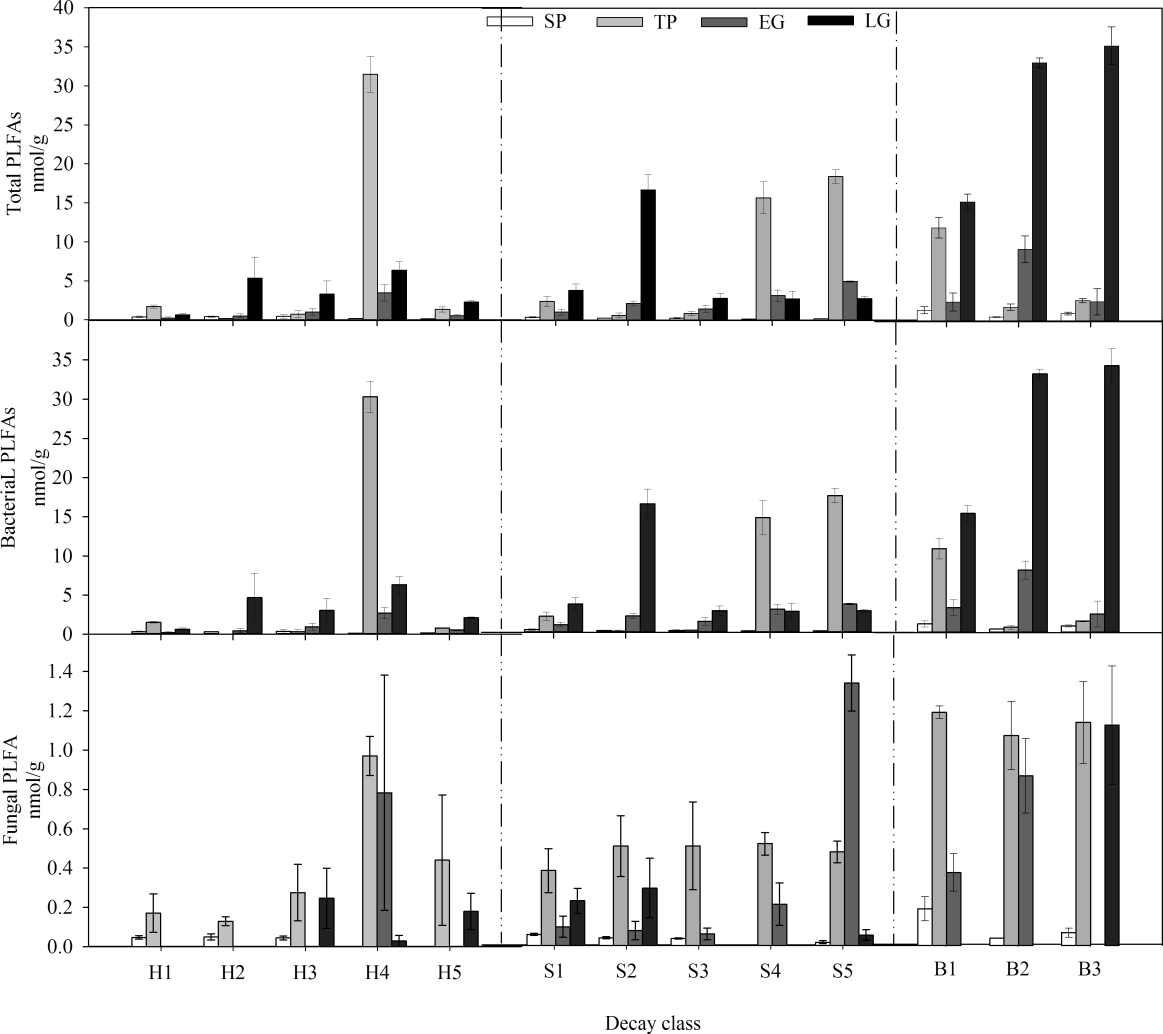
Temporal dynamics of total PLFAs and the sum of bacterial PLFA and fungal PLFA concentrations for fallen log components (heartwood = H, sapwood = S, and bark = B) in different decay classes (decay classes 1–5 for heartwood and sapwood, decay classes 1–3 for bark) in the alpine fir forest of the eastern Qinghai-Tibet Plateau. Bars indicate standard errors.

Both critical period, decay class and the interaction between them had a significant effect on PLFA_tot_ concentrations (Table 2). The factor of critical period had the strongest influence on PLFA_tot_ concentrations, whereas decay class was the weakest among the factors. Trace concentrations of PLFA_tot_ were detected in SP (ranging from 0.163 nmol·g^-1^ to 1.373 nmol·g^-1^) (Fig 4), which was also the lowest concentration in the course of one year. However, the highest PLFA_tot_ concentrations varied with decay class. No significant difference was observed between log components. The highest PLFA_tot_ concentrations in fallen logs of DC2-3 were detected in LG and ranged from 7.34 to 66.06 times the values in SP, while the highest PLFA_tot_ concentrations in fallen logs of DC1 and DC4-5 were detected in TP, ranging from 0.46- (DC1) to 163.31-fold (DC4-5) the variation in SP. The PLFA_tot_ concentration in bark ranged from 0.500±0.041 to 35.174±2.427 nmol·g^-1^ (average 9.711±3.564 nmol·g^-1^) regardless of treatment, which was substantially greater and more variable than in sapwood (from 0.196±0.012 to 18.441±0.872 nmol·g^-1^, average 4.083±1.281 nmol·g^-1^) and heartwood (from 0.163±0.019 to 31.477±2.301 nmol·g^-1^, average 3.038±1.549 nmol·g^-1^).

**Table 2:** Results of repeated measures of critical period (Period) and decay class (DC) on PLFA_tot_, bacterial, fungal, Gram^+^, Gram^-^ and Micro PLFA biomasses over time. The interaction terms are nested within parentheses.

	Factor	df	F_total_	F_bacteria_	F_fungi_	F_Gram+_	F_Gram_-	F_Micro_	α-diversity	β-diversity
Heartwood	Period	3	64.159***	68.237***	4.877**	20.300***	114.890***	1.829	8.204**	12.291***
	Decay class	4	43.855***	46.542***	3.742*	15.873***	76.908***	1.035	6.253**	8.878**
	Period(Decay class)	12	58.421***	66.517***	1.882	17.915***	104.040***	0.715	10.182***	11.790***
Sapwood	Period	3	79.398***	79.398***	28.587***	5.460**	49.217***	74.602***	5.567**	80.806
	Decay class	4	46.652***	46.652***	7.342**	2.124*	34.498***	24.892***	5.212*	1.005
	Period(Decay class)	12	46.776***	46.776***	11.431***	4.698*	33.641***	21.213***	7.313***	3.113**
Bark	Period	3	412.678***	412.678***	41.292***	1.559	14.614***	1.531	22.568***	3.795*
	Decay class	3	6.734*	6.734*	0.994	0.41	1.252	0.96	4.887	1.857
	Period(Decay class)	9	47.455***	47.455***	13.906***	3.715*	4.329**	1.225	11.674***	1.874

*P<0.05

**P<0.01

***P<0.001.

The absolute bacterial PLFA concentration followed similar patterns as PLFA_tot_ throughout the whole-year incubation (Fig 4, Table 2). The factor of critical period also played the most important role in controlling the fungal PLFA concentration (Table 2). The lowest fungal PLFA concentration was detected in SP (ranging from 0.000 to 0.186 nmol·g^-1^), and no concentration was observed in the samples of DC4-5. The highest fungal PLFA concentration was detected in TP (ranging from 0.129 to 0.970 nmol·g^-1^), except in sapwood of DC5 (Fig 4). The fungal PLFA concentration in decaying bark ranged from 0.000 to 1.186±0.031 nmol·g^-1^ (average 0.502±0.150 nmol·g^-1^) regardless of decay class and critical period and was substantially greater and more variable than in sapwood (from 0.000 to 0.334±0.143 nmol·g^-1^, average 0.243±0.072 nmol·g^-1^) and heartwood (from 0.000 to 0.970±0.100 nmol·g^-1^, average 0.168±0.061 nmol·g^-1^).

### Microbial community structure

Only within various critical periods were there pronounced differences both in the relative abundance of fungal to bacterial and Gram^+^ bacteria to Gram^-^ bacteria (Fig 2). The highest averaged fungal to bacterial abundance and Gram^+^ bacteria to Gram^-^ bacteria abundance were observed in TP and SP, respectively; nevertheless, both the lowest abundances were concurrently detected in LG. At the scale of decay stage, the samples of DC2 and DC1 experienced the highest fungal to bacterial abundance and Gram^+^ bacteria to Gram^-^ bacteria abundance, while the lowest abundances were detected in samples of DC4 and DC2, respectively. Considering the structural components, the lowest fungal to bacterial abundance and Gram^+^ bacteria to Gram^-^ bacteria were both detected in heartwood, whereas the most abundant of fungal to bacteria and Gram^+^ bacteria to Gram^-^ bacteria were observed in sapwood and bark, respectively.

**Fig 5:**
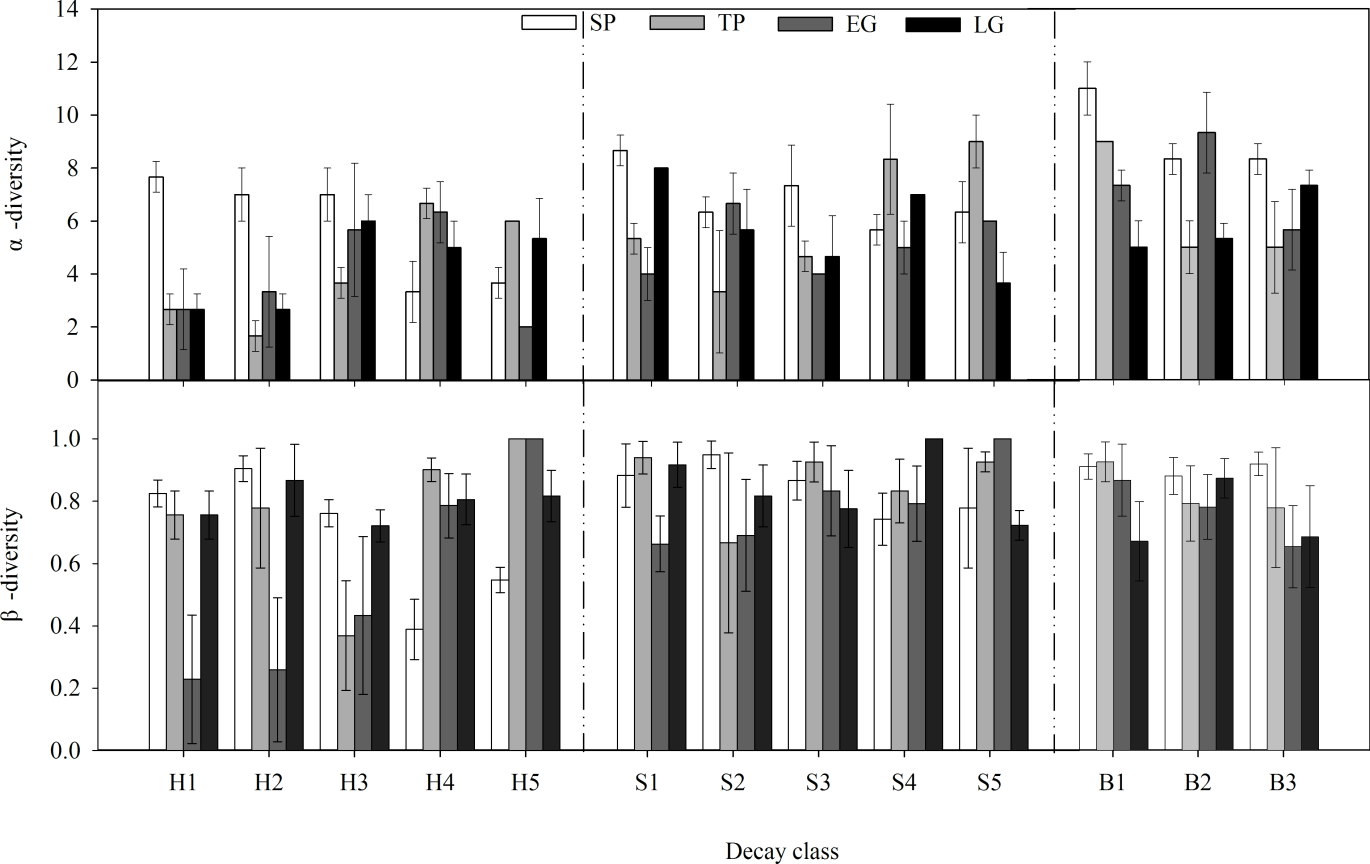
Temporal changes in α- and β-diversity of PLFA monomers for fallen log components (heartwood = H, sapwood = S, and bark = B) in different decay classes (decay classes 1–5 for heartwood and sapwood, decay classes 1–3 for bark) in the alpine fir forest of the eastern Qinghai-Tibet Plateau. Bars indicate standard errors.

Both critical period, decay class and the interaction between them had a significant effect on α-diversity and a less significant effect on β-diversity (Table 2). Pronounced shifts in α- and β-diversity (Fig 5) were observed between SP and TP, and they were greatly dependent on decay class. The highest diversity values for samples of DC1-3 were detected in SP, when the samples of DC4-5 presented the lowest values. Interestingly, the lowest diversity values for samples of DC1-3 were detected in TP, whereas the samples of DC4-5 presented the highest values. The α-diversity in heartwood (4.55±0.43) was significantly lower than that in sapwood (5.98±0.38) and bark (7.22±0.59), while they were not significantly different from one another. β-diversity was less marked between log components (Fig 5).

We analyzed only individual PLFAs with a frequency of occurrence greater than 10% (Table 3), which was also varied with critical periods, decay classes and log components. The absolute advantage PLFA monomers (a-PLFAs, >60%) in SP were 15:0, 16:0, 16:1ω5t, fungal biomarker 18:2ω6,9c, and Gram^-^ biomarker 16:1ω7c. The richest a-PLFA composition was also detected in SP, with succession indexes between 1.50 and 3.50. A significant decrease in frequency for PLFAs 16:1ω5t and 16:1ω7c was detected in TP, while a marked increase for PLFAs 18:1ω7c, 17:0 and 18:3 were detected in heartwood and sapwood. The succession indexes in TP were between 3.25 and 4.75. PLFAs 16:0 and 16:1ω9c occurred most frequently in all log components in EG. PLFAs 16:0 and 18:1ω7c occurred most frequently in heartwood and bark in LG, while PLFAs 16:1ω5t and 16:1ω9c displayed a 100% presence in all sapwood samples.

**Table 3:** Succession index and frequency of single PLFA monomers in decaying fallen log components (heartwood, sapwood, and bark) at each critical period in the alpine fir forest of the eastern Qinghai-Tibet Plateau.—frequency of single PLFA monomers lower than 10%, SP seasonal snow cover period, TP snow thawing period, EG early growing season, LG late growing season.

	Biomarker	Succession index				Frequency			
		SP	TP	EG	LG	SP	TP	EG	LG
Heartwood	i 15:0	2.83	3.63	1.00	-	40.00%	53.33%	20.00%	-
	a 15:0	-	-	4.00	-	-	-	6.67%	-
	16:0	1.91	3.33	3.09	3.00	73.33%	60.00%	73.33%	80.00%
	17:0	2.83	4.00	1.50	1.80	40.00%	6.67%	13.33%	33.33%
	a17:0	2.40	-	1.00	1.00	33.33%	-	6.67%	13.33%
	16:1w7c	1.33	1.00	2.25	2.00	60.00%	6.67%	53.33%	60.00%
	15:0	2.42	3.56	2.22	4.00	80.00%	60.00%	60.00%	6.67%
	16:1w5t	2.60	1.00	2.40	4.00	100.00%	6.67%	33.33%	46.67%
	i17:0	-	-	-	-	-	-	-	-
	16:1w9c	1.38	2.91	3.33	3.33	53.33%	73.33%	60.00%	60.00%
	18:1w7c	2.00	4.00	2.60	2.60	6.67%	20.00%	33.33%	100.00%
	18:2w6,9c	1.33	2.71	4.00	3.00	60.00%	93.33%	13.33%	26.67%
	18:3	1.67	4.75	1.50	4.00	20.00%	26.67%	13.33%	6.67%
	20:4	4.00	1.00	1.00	-	6.67%	6.67%	13.33%	-
	cy17:0	-	-	-	-	-	-	-	-
	cy19:0	-	-	-	-	-	-	-	-
Sapwood	i 15:0	2.73	2.60	3.33	-	73.33%	100.00%	20.00%	-
	a 15:0	4.50	-	4.60	1.00	26.67%	-	33.33%	6.67%
	16:0	1.91	2.69	2.60	2.09	73.33%	86.67%	100.00%	73.33%
	17:0	1.00	4.17	2.00	2.86	13.33%	40.00%	13.33%	46.67%
	a17:0	1.00	4.00	2.00	2.50	20.00%	33.33%	13.33%	40.00%
	16:1w7c	2.60	3.33	1.67	1.20	100.00%	20.00%	20.00%	33.33%
	15:0	2.31	3.44	2.85	1.67	86.67%	60.00%	86.67%	20.00%
	16:1w5t	2.60	-	1.29	2.60	100.00%	-	46.67%	100.00%
	i17:0	4.00	-	-	-	6.67%	-	-	-
	16:1w9c	2.60	3.33	2.85	2.60	100.00%	60.00%	86.67%	100.00%
	18:1w7c	1.00	3.25	5.00	2.43	13.33%	53.33%	20.00%	93.33%
	18:2w6,9c	2.00	2.60	2.82	2.43	73.33%	100.00%	73.33%	46.67%
	18:3	-	4.50	-	4.00	-	40.00%	-	20.00%
	20:4	-	1.00	-	-	-	20.00%	-	-
	cy17:0	-	-	-	-	-	-	-	-
	cy19:0	-	-	-	-	-	-	-	-
Bark	i 15:0	2.17	1.88	2.00	1.00	66.67%	88.89%	55.56%	11.11%
	a 15:0	1.50	1.00	2.50	3.00	22.22%	11.11%	22.22%	11.11%
	16:0	2.00	2.00	2.00	2.00	100.00%	88.89%	100.00%	100.00%
	17:0	2.00	1.50	1.67	2.00	77.78%	44.44%	33.33%	22.22%
	a17:0	2.00	1.40	1.40	2.00	88.89%	55.56%	55.56%	22.22%
	16:1w7c	2.00	1.00	2.00	2.33	100.00%	33.33%	66.67%	66.67%
	15:0	2.00	1.88	1.71	2.40	100.00%	88.89%	77.78%	55.56%
	16:1w5t	1.88	-	2.75	2.00	88.89%	-	44.44%	66.67%
	i17:0	-	3.00	-	3.00	-	22.22%	-	11.11%
	16:1w9c	2.00	2.00	2.00	1.88	100.00%	66.67%	100.00%	88.89%
	18:1w7c	1.00	1.00	1.71	2.00	33.33%	33.33%	77.78%	100.00%
	18:2w6,9c	2.00	2.00	1.50	3.00	100.00%	100.00%	66.67%	33.33%
	18:3	1.00	-	2.00	-	11.11%	-	22.22%	-
	20:4	1.00	-	3.00	-	33.33%	-	22.22%	-
	cy17:0	-	-	-	-	-	-	-	-
	cy19:0	-	-	-	-	-	-	-	-

PLFA 18:2ω6,9c was the most frequent monomer in TP, with a frequency of 100% in sapwood and bark and 93.33% in heartwood. The succession index of 18:2ω6,9c was between 1.33 and 4.00 in heartwood, whereas it was more clustered in sapwood and bark (1.50–3.00) (Table 3). Both the concentration and frequency of 18:2ω6,9c in all log components were higher in SP and TP than in EG and LG.

### NMDS analysis

The structure of microbial taxonomic groups in heartwood had the highest homogeneity, while the structure of microbial taxonomic groups in sapwood and bark showed high variation (Fig 3). The microbial communities in SP were significantly different from the ones in LG along axis 1 and the ones in TP along axis 2 in sapwood and bark, overall. The microbial communities in SP tended to cluster along axis 1 ignoring the variation in decay class, while the microbial community in TP tended to cluster separately from each other in three groups (i.e., DC1, DC2-3, and DC4-5). The group formed by DC2-3 was closer to the SP group.

A joint plot constructed using taxonomic markers suggested that the microbial communities in SP and EG tended to have lower concentrations of all taxonomic markers, that microbial communities in TP tended to have higher concentrations of fungi and Gram^+^, and that microbial communities in LG tended to have higher concentrations of bacteria and Gram^-^, which was consistent with the results of previous analyses (Fig 4). An examination of the NMDS loadings showed that bacteria and Gram^-^ contributed mainly to axis 1, while Gram^+^ and fungi contributed mainly to axis 2.

#### Correlations

The PLFA_tot_ biomasses in all log components were positively correlated with log moisture contents in TP (Table 4). α-diversity was negatively correlated with log moisture content in SP and was positively correlated with log moisture in TP. β-diversity was negatively correlated with log moisture content in SP.

**Table 4:** Correlation coefficients between total PLFA biomass (PLFA_tot_) and α-diversity and β-diversity indexes with the moisture contents of fallen log components (heartwood, sapwood and bark) in each critical period. SP snow cover period, TP snow thawing period, EG early growing season, LG late growing season.

	Heartwood			Sapwood			Bark		
	PLFA_tot_	α-diversity	β-diversity	PLFA_tot_	α-diversity	β-diversity	PLFA_tot_	α-diversity	β-diversity
SP	-2.12	-0.777**	-0.686**	-0.315	-0.223	-0.525*	0.465	-0.152	-0.300
TP	0.733**	0.633*	-0.169	0.643**	0.708**	0.350	0.781**	0.373	0.012
EG	0.425	0.003	0.347	0.183	0.091	-0.145	0.387	0.565*	-0.309
LG	-0.046	-0.145	-0.194	-0.057	-0.241	-0.433	0.960**	0.158	-0.279

*P<0.05

**P<0.01.

## Discussion

### The effects of critical period on microbial community structure

We found pronounced effects of critical period on microbial biomass and the microbial community, as assessed by in situ incubation of fallen logs at various decay stages. Our data indicated that the seasonal pattern of microbial biomass was largely controlled by moisture content, especially when met with extremely low temperatures and large temperature fluctuations (Table 4). Continuous low temperatures and temperature fluctuations are known to be fatal to microbes, but in this study, like most studies conducted in soil and litter decomposition [32,33], abundant log microbes were also detected in SP. Similarly, a pronounced increase in microbial biomass was detected in TP as well. Apparently, we cannot attribute this result to the snow cover, as previous studies have, due to the large volume of logs. Actually, most of the logs were nearly exposed to the air, especially in the closed canopy, when the deepest snow cover was formed in the forest gap [34]. Thus, our results hint that such fluctuation could be linked to the structural composition, which consisted of chemical components, porosity, and moisture. The texture of fir wood is tighter, with high total contents of lignin and cellulose ranging from 49% to 64% [35]. Moreover, the lowest moisture content (Table 1) in SP contributed to an excellent thermal insulation property of logs. The rich microbial biomass in TP also suggested an excellent buffer of environmental variation (Fig 4) in fallen logs. Overall, the seasonal dynamics of microbial community structure on fallen logs (Figs 3 and 4) were seemingly consistent with previous literature about soil and litter in high-frigid regions. What the difference is the mechanism that log shelters microbial diversity by physical insulation, which could possibly elevate the resistance of the microbial community to a fluctuating environment [36].

In line with a study conducted in tundra soils [37], a high abundance of fungal was detected in winter, with an especially significant high biomass and relative abundance of fungal to bacteria in SP. Norros et al. [38] concluded that extremely low temperatures can be fatal to fungal spores, which was inconsistent with our study. Such a difference could be linked to the buffering of environmental variation of fallen logs as well, whereas most previous studies concentrated on water-holding capacity and cooling capacity, especially in drought and hot climate ecosystems. This finding emphasizes the need to maintain the existence of fallen logs in high-frigid ecosystems with decreasing snow cover under climate change.

As a result of natural selection, those surviving microbes from SP, such as biomarkers 16:1ω7c and 16:1ω5t, must be more cold stable but susceptible to snow melting. Studies conducted in tundra soils have also demonstrated that PLFA monomers 16:1ω7c and 16:1ω5t increased significantly after 11 weeks of incubation under in situ conditions [39]. Conversely, PLFAs 18:1ω7c, 17:0 and 18:3 were more tolerant of snow melting but sensitive to freezing climate. Consequently, PLFAs 16:1ω7c and 16:1ω5t and PLFAs 18:1ω7c, 17:0, and 18:3 contributed greatly to the shifts in α- and β- diversity from SP to TP for samples of DC2-3 and samples of DC4-5, respectively.

The fact that seasonal snow cover and freeze-thaw events regulate the decomposition process in high altitude and latitude ecosystems has been highlighted in many studies [24,34,40,41], but few studies have concentrated on fallen logs. According to our previous work, the degradation of cellulose and lignin in winter accounted for more than 50% of two years of foliar litter degradation [24,34]. A study of wood [42] also showed that the physical structure of wood can be extremely sensitive to repeated freezing and thawing, which may also promote the decay process in high-latitude and -altitude regions. The gradually increased microbial biomass in the posterior seasons (Fig 4) may partially suggest improved nutrient levels and, thus, a relatively high decomposition rate. Although the harsh climatic conditions reduced the total microbial biomass, the highest F/B and Gram^+^/Gram^-^ values hint that the resistant components of wood, such as foliar litter, are mainly degraded in SP [34]. The moisture content of fallen logs has been shown to have a positive relationship with respiration in temperate forests [17,43], and through that process over 75% of log carbon is lost [44]. Thus, our results, combined with those studies, suggest that EG should be an important critical period for carbon loss, and microbial organisms contribute significantly to this process.

### The effects of substrate quality on microbial community structure

A smaller but significant difference in taxonomic groups between TP and other critical periods was observed in our study (Fig 3). Samples in TP were not so clustered; in contrast, they were scattered roughly by decay stages. The results indicated a serial seasonal transition from winter to growing season and that some factors (i.e., substrate quality) other than climate conditions controlled the seasonal changes in the microbial community [45,46].

The quality of decaying logs can influence the nature of the microbial community, including its biomass, composition, and function [16,47]. The composition of the microbial community can, in turn, influence the course of decomposition and the chemical changes in logs during decomposition [3,48]. Several potential mechanisms may explain why there was a stronger effect on microbial community composition than on microbial biomass between decay classes in our study. One is the poor substrate availability with a high ratio of C/N throughout the entire decay process, making it difficult to support higher biomass at all decay classes. Another is the coniferous log leachate, which can be very acidic and rich in polyphenols and thus bacteriostatic as well. Finally, the changes in composition of recalcitrant carbon compounds and labile carbon varied with decay class, which could possibly stimulate enzyme production and alter the trophic interactions among organisms rather than microbial biomass.

A significantly higher Gram^+^ bacteria to Gram^-^ bacteria abundance was observed at DC1 in our study, similar to that in soil [49], indicating that Gram^+^ bacteria were among the first colonizers to degrade deadwood once it contacted forest soils. The substantial nonstructural components (such as starch, soluble carbohydrates and proteins) from cell contents and nutrients originating from organic compounds [12] tend to stimulate the activity of Gram^+^ bacteria. Consequently, a high mass loss efficiency was resulted and the deadwood was more susceptible to Gram^-^ bacteria [50] (Fig 2). Similarly, Hoppe et al. [51], who studied the sprawling bacterial diversity and community in deadwood using 16S rDNA pyrosequencing, also detected *Methylovirgula*, a typical Gram^-^ bacteria, as the most abundant OUTs during the wood decay process. In line with a previous study [4], fungi were also found among the first colonizers to degrade deadwood. Moreover, our study revealed a peak of fungal colonization in DC2 and a decrease in late decay classes (Fig 2). A long-term CWD addition experiment also detected a significantly higher fungi-to-bacteria ratio and utilization efficiency of phenol in soils with doubled wood treatment [52]. Together with studies of the proximity effects of adding woody debris to the forest floor, the above data all suggest that maintaining a certain amount of deadwood on the forest floor can influence the decomposition process and utilization efficiency of compounds, thus regulating the ecological process.

Our study revealed significantly higher PLFA biomass and diversity and stronger seasonal fluctuations in bark than in wood. The bark of fallen logs must be the first component to be affected by ongoing events caused by climate change. The existence of bark seems to contribute strongly to resistance to decomposition because of its peculiar anatomical structure and chemical composition [53,54]. Additionally, we not only observed that heartwood samples experienced the most stable seasonal composition of the taxonomic groups but also detected significantly higher F/B and Gram^+^/Gram^-^ values, which all indicate a greater decomposition efficiency for heartwood. Shorohova and Kapitsa [54] compared the decomposition rates of bark and wood, and they found diverse rates varying among tree species, diameters, and fragmentation. All these results indicate that studies considering heartwood, sapwood, and bark together as one substrate may produce a less accurate portrayal of microbial dynamics.

### The comprehensive effects of critical period and substrate quality on microbial community structure

Some of the most obvious changes in diversity were detected in SP and TP between different decay classes, which we considered to primarily result from the combined effects of hydrothermic factors and nutrient availability. We confirmed that the high moisture-holding capacity [55] and specific surface area [2] of late decay class logs would have a strong suppressive effect on microbial biomass and diversity in SP, when the air temperature is always below the freezing point. In contrast, early decay class logs played a crucial role in sheltering microbial diversity in SP. As temperature increased in TP, the activity of all microbes in all logs was enhanced. However, the microbial community, especially in late decay class logs, transformed sensitively from SP to TP with the warming air temperature. Alternately, the activated microbial community could die rapidly during events with intense warming and subsequent low temperatures.

The high microbial PLFA richness in the samples of DC1-3 played an important role in sustaining microbial composition stability under changing environments [56]. We confirm that, in addition to the increased temperature, the burst of microbial diversity and biomass in samples of DC4-5 mainly resulted from (1) the broad surface area, which sustained the growth of microbes; (2) high moisture-holding capacity, which was also demonstrated to be positively related to PLFA_tot_ biomass and α-diversity in this period (Table 4); and (3) the frequent freeze-thaw events in SP, which could destroy the chemical structure of lignin and N-lignin complexes [57] in samples of DC4-5, resulting in a quantity of organic and dissolved organic carbon released during melting. As described above, although microbial activity in late decay class logs was temporarily depressed in SP, microbial biomass and diversity were subsequently promoted in the following periods with sufficient space and resources. Numerous studies [36,58,59] have described the positive effects of diversity on the functioning of communities arising from complementary resource use and facilitation. We assume that high biomass and diversity in TP could also promote the decay rates of fallen logs. However, Fukami et al. [60] and Jousset et al. [61] argued that the enhanced competition for space and resources in species-rich communities can have a negative impact on the decomposition rate by reducing fungal growth and counteracting facilitative interactions.

We discovered not only Gram^+^ bacteria were better adapted to freezing than Gram^-^ bacteria but also that Gram^-^ bacteria were more competitive in the degradation of nutritional substrates than Gram^+^ bacteria. With the input of fresh plant residuals, invertebrate activity [23] also greatly improved the substrate availability of deadwood [62] in LG, which seemed to be the most favorable period for microbes with significantly lower relative abundance of fungal to bacteria and Gram^+^ bacteria to Gram^-^ bacteria values.

## Conclusions

Significant changes in microbial biomass and diversity between SP and TP were discovered in both early decay class and late decay class fallen logs, the latter of which was more sensitive to seasonal fluctuations. Although decay class had a less pronounced effect on the microbial community than critical period, decay class influenced microbial community structure more than microbial biomass. The result that seasonal decay mode varied with structural components strongly implied that studies treating heartwood, sapwood and bark as a whole are less accurate. Our results also imply that the decomposition of woody debris on the forest floor alters the ecosystem process by influencing woody debris and beneath the soil.

We confirm that the microbial community compositions of fallen logs were affected by both physicochemical wood properties and environmental factors. It was also varied with critical periods, but we were unable to quantify the contribution of each factor in each decay class and critical period subject to the research progress. Fortunately, the responses of taxonomic groups and PLFA biomarkers to a given decay class and critical period can indirectly reflect stress from nutrients and the environment. The results presented in this study elucidate the seasonal dynamics of fallen log-dwelling organisms in various decay classes and the potential response of the microbial community to climate change. Moreover, the wide temporal fluctuations emphasize the need for sampling both seasonal and annually to fully interpret the dynamics of microbial populations during wood decay.

## Supporting information

S1 Table Seasonal changes in the moisture content of three fallen log components. (XLSX)

S2 Table Dynamics of daily mean temperature in the atmosphere at the study site during the study period. (XLSX)

S3 Table Temporal dynamics of total PLFAs and bacterial PLFA and fungal PLFA concentrations in fallen logs. (XLSX)

S4 Table The occurrence of PLFA signature in fallen logs. (XLSX)
